# A global assessment of the impact of school closure in reducing COVID-19 spread

**DOI:** 10.1098/rsta.2021.0124

**Published:** 2022-01-10

**Authors:** Joseph T. Wu, Shujiang Mei, Sihui Luo, Kathy Leung, Di Liu, Qiuying Lv, Jian Liu, Yuan Li, Kiesha Prem, Mark Jit, Jianping Weng, Tiejian Feng, Xueying Zheng, Gabriel M. Leung

**Affiliations:** ^1^ WHO Collaborating Centre for Infectious Disease Epidemiology and Control, School of Public Health, LKS Faculty of Medicine, The University of Hong Kong, Hong Kong; ^2^ Laboratory of Data Discovery for Health (D^2^4H), Hong Kong Science Park, New Territories, Hong Kong; ^3^ Department of Communicable Diseases Control and Prevention, Shenzhen Center for Disease Control and Prevention, Shenzhen 518055, People's Republic of China; ^4^ The First Affiliated Hospital of USTC, Division of Life Science and Medicine, University of Science and Technology of China, Hefei, People's Republic of China; ^5^ Clinical Research Hospital (Hefei) of Chinese Academy of Science, Hefei, People's Republic of China; ^6^ Anqing Hospital Affiliated to Anhui Medical University (Anqing Municipal Hospital), Anqing, People's Republic of China; ^7^ Centre for Mathematical Modelling of Infectious Diseases, Department of Infectious Disease Epidemiology, London School of Hygiene and Tropical Medicine, London, UK

**Keywords:** COVID-19, SARS-CoV-2, school closure, epidemiology, susceptibility, infectiousness

## Abstract

Prolonged school closure has been adopted worldwide to control COVID-19. Indeed, UN Educational, Scientific and Cultural Organization figures show that two-thirds of an academic year was lost on average worldwide due to COVID-19 school closures. Such pre-emptive implementation was predicated on the premise that school children are a core group for COVID-19 transmission. Using surveillance data from the Chinese cities of Shenzhen and Anqing together, we inferred that compared with the elderly aged 60 and over, children aged 18 and under and adults aged 19–59 were 75% and 32% less susceptible to infection, respectively. Using transmission models parametrized with synthetic contact matrices for 177 jurisdictions around the world, we showed that the lower susceptibility of school children substantially limited the effectiveness of school closure in reducing COVID-19 transmissibility. Our results, together with recent findings that clinical severity of COVID-19 in children is lower, suggest that school closure may not be ideal as a sustained, primary intervention for controlling COVID-19.

This article is part of the theme issue ‘Data science approach to infectious disease surveillance’.

## 1. Introduction

As the COVID-19 pandemic began to hit different locations during the first wave, many jurisdictions around the world had pre-emptively implemented school closure to suppress and mitigate its local spread [1,2]. The UN Educational, Scientific and Cultural Organization (UNESCO) estimated that during April, May and June 2020, around 1.6, 1.2 and 1.1 billion (90%, 68% and 62%) of enrolled learners around the world were affected by country-wide or localized school closure (https://en.unesco.org/covid19/educationresponse). Such swift and widespread adoption of prolonged school closure (which lasted weeks to months) was largely predicated on the premise that school children would be a core group for COVID-19 transmission as they are for influenza [2]. As countries are reviewing strategies for social and economic resumption, there is a need to evaluate the rationale and effectiveness of school closure against COVID-19 [3].

Reducing contacts made at school has been effective in controlling influenza because (i) children are biologically more or equally as susceptible to infection compared with adults; (ii) children have more contacts with other children at school than adults have with their peers; and (iii) children are less vigilant about personal infection control (e.g. hand hygiene) compared with adults [4,5]. Indeed, previous epidemiological studies in different populations have shown that the reproductive number of seasonal influenza and A/H1N1 pandemic influenza in 2009 (H1N1pdm09) was significantly reduced during school closure or holidays (table 1). While (ii) and (iii) are generally true, it is not clear whether (i) is valid for COVID-19. Although an early analysis of contact tracing and surveillance data from Shenzhen suggested that age had no effect on susceptibility to COVID-19 infection [13], more recent studies indicated that children might only be 35–50% as susceptible as adults [14–16].

**Table 1.** Comparison between our results and published estimates of reduction in reproductive number for H1N1pdm09 and seasonal influenza during school closure and holidays. In these comparisons, school contacts were assumed to be reduced by 60% during school closure and holidays.

	published estimates	our estimates
H1N1pdm09 in the UK during summer holidays [6]	35% (30–40%)	37%
H1N1pdm09 in Hong Kong during closure of kindergartens and primary schools [7]	13% (10–15%)	16%
H1N1pdm09 in Hong Kong during summer holidays [7]	35%	37%
H1N1pdm09 in India during summer holidays [8]	14–27%	26%
H1N1pdm09 in China during school holidays [9]	37% (28–45%)	33%
seasonal influenza in France during summer holidays in 1985–2006 [10]	13–17%	17% if no age effect
		35% if H1N1pdm09-like
seasonal influenza B in Hong Kong during closure of kindergartens and primary schools in 2018 [11]	16% (10–26%)	7% if no age effect
		16% if H1N1pdm09-like
seasonal influenza in South Korea during school holidays in 2014–2016 [12]	6–23%	17% if no age effect
		38% if H1N1pdm09-like

On the other hand, there are considerable downsides to prolonged school closure, especially when recent seroprevalence studies suggest that there is not yet sufficient population immunity to avert subsequent waves of infection [17,18] until a safe and effective vaccine becomes widely available to the majority of the global population, and that could be a year or longer away. Governments have a duty of care to school-aged children, both for the infection risk of congregating in schools and also for their developmental and learning needs if schools are indefinitely suspended. School-aged children with special needs are particularly adversely affected. Of course, parental duties for childcare have been non-trivial and are a cause of widening inequity by socioeconomic position.

We aim to assess the potential impact of school closure in reducing the spread of COVID-19 in different parts of the world. To this end, we first inferred the effect of age on COVID-19 susceptibility and infectiousness using detailed surveillance and contact tracing data from Shenzhen and Anqing, China (the raw data from Shenzhen was the same as that in [13]) as well as published estimates from Guangzhou and Hunan, China [15,16]. We then parametrized our previous age-structured transmission model [19] with the inferred epidemiological parameters to simulate the epidemiological impact of school closure in an otherwise unmitigated epidemic in 177 jurisdictions around the world for which contact matrices have been synthesized [20]. To gauge the relative effectiveness of school closure in comparison with other non-pharmaceutical interventions (NPIs), we estimated the reduction in infectious contacts made in non-school settings that would be needed in order to achieve the reduction in transmissibility or schoolchildren infection attack rate (IAR) conferred by school closure.

## 2. Methods

### (a) Data on within-household transmission

**Figure 1.**
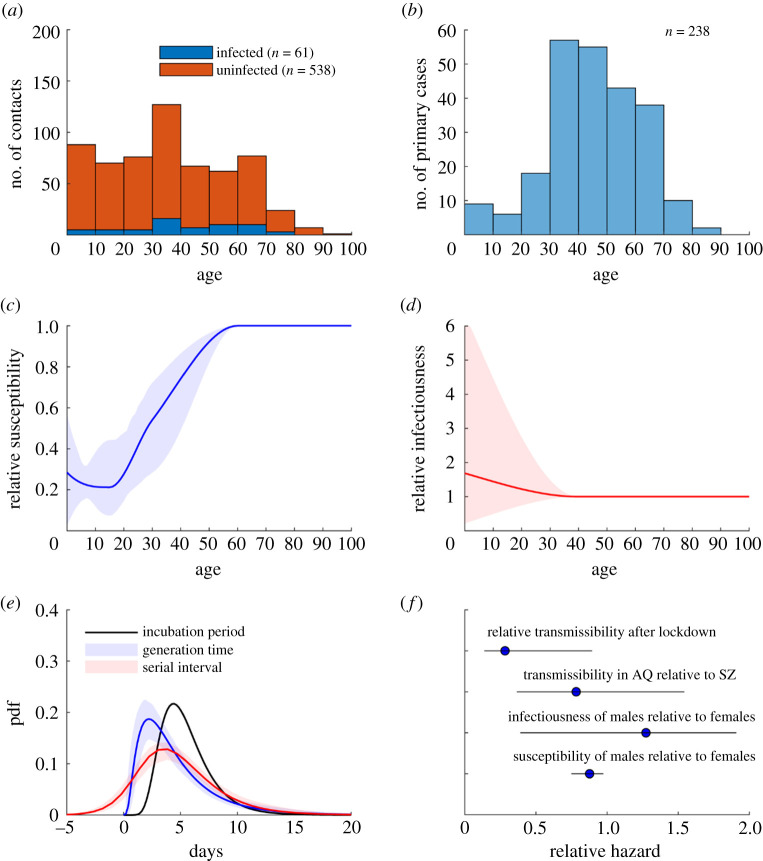
Within-household transmission dynamics of COVID-19 among 182 clusters in Shenzhen and Anqing. Key assumptions included the incubation period distribution was lognormal with median 5.1 days and mean 5.5 days [21]; susceptibility was the same for those aged 60 or above; infectiousness was the same for those aged 40 or above. (*a*,*b*) Age distribution of household contacts (stratified by infection outcome) and primary cases. (*c–e*) Age-specific susceptibility, age-specific infectiousness, generation time distribution and serial interval distribution. Lines and shades correspond to MAP estimates and 95% Crls, respectively. (*f*) Susceptibility and infectiousness of males relative to females; transmissibility in Anqing relative to Shenzhen; and relative transmissibility after versus before Wuhan lockdown. Circles and bars correspond to MAP estimates and 95% CrIs, respectively. (Online version in colour.)

We analysed within-household transmission of COVID-19 in Shenzhen and Anqing based on the COVID-19 line-lists and outbreak investigation records collated and compiled by the Shenzhen and Anhui Centers for Disease Control and Prevention. Details about these databases have been previously reported (figure 1*a,b*) [13,22]. For this study, we first identified cases who were almost certainly infected outside their household based on their travel and contact history. We then inferred the membership of their households based on outbreak investigation records. After excluding households with more than eight members (because they were rare and we were relatively uncertain whether these clusters were bona fide households), our dataset comprised a total of 182 household clusters (147 from Shenzhen reported between 1 January and 8 March 2020, and 35 from Anhui reported between 9 January and 20 February 2020; see electronic supplementary material, figures S1–S13) with 238 primary/co-primary cases and 599 household contacts.

A close contact was defined to be infected if any of his/her SARS-CoV-2 RT-PCR tests were positive during isolation (at home or designated facilities). Since 20 January 2020, in both Shenzhen and Anqing, individuals who fulfilled one of the following criteria were traced: (1) resided in or had travel history to Wuhan or Hubei in the past 14 days; (2) had exposure to wild animals in the past 14 days; (3) had influenza-like or COVID-19 like symptoms such as fever, dry cough, dyspnoea and diarrhoea and (4) had close contacts with confirmed or suspected COVID-19 patients within 14 days before and after their symptom onset. Individuals who fulfilled any of the above criteria were identified by the community or local CDC staff in person or by telephone and were placed under close epidemiological investigation. They were isolated at home (before 24 January 2020) or designated facilities (on or after 24 January 2020) for medical surveillance for 14 days and had a SARS-CoV-2 RT-PCR test upon onset of symptoms or every 3 days if there were no symptoms. Individuals were admitted to designated hospitals once their SARS-CoV-2 RT-PCR test was positive.

### (b) Household transmission model

We developed a transmission model to simulate the spread of COVID-19 within households as follows. Given that during the study period there were only 417 and 83 cases of COVID-19 in Shenzhen and Anqing, respectively, and that contact tracing was very extensive, the probability of multiple importations within a household (i.e. some of the non-primary cases were infected via community transmission that were not identified by contact tracing) was likely to be negligible in our dataset. As such, we assumed that among household contacts, the risk of community transmission was negligible compared with the force of infection exerted by the primary/co-primary cases. Following previous household transmission modelling studies for H1N1pdm09, we assumed that interactions, case ascertainment and infection prevention measures were homogeneous among members within households (i.e. did not depend on age) [23,24]. We assumed that the probability of case ascertainment was 100% in the base case and 70% in the sensitivity analyses (electronic supplementary material, figure S14).

We inferred the following parameters by fitting the model to the data: (i) age-specific susceptibility to infection; (ii) age-specific infectiousness; (iii) relative susceptibility and infectiousness of males compared with females; (iv) baseline transmission rate for each household size; (iv) the generation time distribution; (v) the transmission rate in Anqing relative to Shenzhen; and (vi) changes in within-household transmission rate after nationwide lockdown measures were implemented. We used *θ* to denote the set of parameters subject to statistical inference.

#### (i) Infectiousness profile and generation time distribution

Suppose individual *j* was infected by individual *i*. We used $f_{\text{inc}}(\cdot )$ to denote the probability density function (pdf) of the incubation period and assumed that their incubation periods were independent and denoted them by *X_i_* and *X_j_*, respectively. Let *Y* be the time it took individual *i* to infect individual *j* after the former had become infected (i.e. the generation time for this pair). We assumed that the pdf of *Y* was dependent on *X_i_* as follows:

$$P(Y=y\text{|}X_{i}=x)=f_{\text{gen}}(y\text{|}\theta ,x)=\left\{ \begin{array}{ll} 0 & \text{if }y<\mu_{\text{inf}}x;\\ g(y-\mu_{\text{inf}}x|a_{\text{inf}},b_{\text{inf}}) & \text{if }y\geq \mu_{\text{inf}}x, \end{array}\right.$$

where $g(\cdot |a_{\text{inf}},b_{\text{inf}})$ was the gamma pdf with shape *a*_inf_ and scale *b*_inf_, *μ*_inf_*x* was the time at which individual *i* became infectious which was assumed to increase linearly with the incubation time *x*. We assumed that the time it took infectiousness to peak after the infected individual had become infectious was *ρ*_inf_*x* (such that *b*_inf_ = *ρ*_inf_*x*/(*a*_inf_ − 1)). The parameters (*μ*_inf_, *a*_inf_, *ρ*_inf_) were included in *θ* for statistical inference. The unconditional pdf of the generation time for individuals *i* and *j* was thus

$$\begin{array}{rl} f_{\text{gen}}(y\text{|}\theta ) & =P(Y=y)=\int_{0}^{\infty }P(Y=y\text{|}X_{i}=x)f_{\text{inc}}(x)\;\text{d}x\\ & =\int_{0}^{\infty }f_{\text{gen}}(y\text{|}\theta ,x)f_{\text{inc}}(x)\text{d}x. \end{array}\}$$

The pdf of the serial interval for individuals *i* and *j*, namely *Z* = *Y* + *X_j_* − *X_i_*, was

$$\begin{array}{rl} f_{SI}(z\text{|}\theta ) & =P(Z=z)=P(Y+X_{j}-X_{i}=z)\\ & =\int_{0}^{\infty }P(Y+X_{j}=z+x\text{|}X_{i}=x)f_{inc}(x)\;\text{d}x, \end{array}\}$$

where

$$\begin{array}{rl} P(Y+X_{j}=z+x\text{|}X_{i}=x) & =\int_{0}^{z+x}P(X_{j}=z+x-u\text{|}X_{i}=x)f_{gen}(u|\theta ,x)\;\text{d}u\\ & =\int_{0}^{z+x}f_{inc}(z+x-u)f_{gen}(u|\theta ,x)\;\text{d}u. \end{array}$$

We used $F_{SI}(\cdot \text{|}\theta )$ to denote the pdf of the serial interval. In the base case, we assumed that the incubation period distribution was lognormal with median 5.1 days and mean 5.5 days (which corresponded to a coefficient of variation of 0.4) [21]. In the sensitivity analyses, we perturbed the mean incubation period by ±1 day while maintaining the same coefficient of variation (electronic supplementary material, figure S14).

#### (ii) Statistical inference of transmission parameters

The overall likelihood function was

$$L(\theta )=\left(\underset{k}{\prod }L_{k}(\theta )\right)L_{SI}(\theta ),$$

where *L_k_*(*θ*) was the likelihood function for cluster *k* and

$$L_{SI}(\theta )=\underset{j\in A}{\prod }f_{SI}(s_{j}\text{|}\theta )\underset{j\in B}{\prod }F_{SI}(s_{j}^{U}\text{|}\theta )-F_{SI}(s_{j}^{L}\text{|}\theta ),$$

was the likelihood function for the serial interval data collected from outside Anqing and Shenzhen. Here, *s_j_* was the serial interval data point for infector–infectee pair *j*, and $s_{j}^{L}$ and $s_{j}^{U}$ were the corresponding lower-bound and upper-bound if there was uncertainty in that data. *A* and *B* referred to the sets of serial interval data without and with uncertainty, respectively (see electronic supplementary material, table S2). The likelihood function *L_k_*(*θ*) was formulated as follows.

We partitioned each cluster *k* into three sets of individuals:

— Set *A_k_* comprised all the primary cases (i.e. those who were infected outside and introduced the infection into the cluster).
— Set *B_k_* comprised all members who were ascertained to be infected during the cluster outbreak initiated by the primary cases in *A_k_*.
— Set *C_k_* comprised all other members in the cluster.

Let *n_k_* be the size of cluster *k*, *a_i_* and *g_i_* be the age and sex of individual *i*, and *T_i_* and *O_i_* be the time of infection and time of symptoms onset if he/she was infected (i.e. *i* ∈ *A_k_* or *i* ∈ *B_k_*), respectively. Our aim was to infer the following epidemiological characteristics of COVID-19 (the corresponding parameters that were included in *θ* for statistical inference are indicated; see below for their detailed formulation):

1. The effect of age on susceptibility to infection (*α*_sus_(0), *α*_sus_(15) and *α*_sus_(30)).
2. The effect of age on infectiousness (*α*_inf_(0)).
3. The effect of sex on susceptibility to infection (*δ*_sus_(male)).
4. The effect of sex on infectiousness (*δ*_inf_(male)).
5. Transmission rate for cluster with sizes *n* = 2, … , 8 (*β*(2), … , *β*(8)).
6. The relative within-household transmissibility of COVID-19 in clusters from Anqing compared to that from Shenzhen (*ω_AQ_*).
7. The relative within-household transmissibility of COVID-19 after lockdown measures were implemented in Wuhan on Jan 23 followed by all major Chinese cities shortly after (*σ*).
8. The pdf of the generation time (*μ*_inf_, *a*_inf_, *ρ*_inf_; see the previous subsection for the formulation of the generation time pdf).

Based on the travel and contact history of the infected cases, outbreak investigators were able to specify a range for their time of infection. We denoted this range by [*T*_min,*i*_, *T*_max,*i*_] for individual *i* ∈ *A_k_* or *i* ∈ *B_k_*, i.e. *T*_min,*i*_ ≤ *T_i_* ≤ *T*_max,*i*_. The likelihood for cluster *k* conditioned on the time of infection of its primary cases (i.e. *T_i_*, *i* ∈ *A_k_*) was formulated as follows:

$$L_{k}(\theta |T_{i},i\in A_{k})=\overset{3}{\underset{m=1}{\prod }}L_{k,m}(\theta |T_{i},i\in A_{k}),$$

where

1. $L_{k,1}(\theta |T_{i},i\in A_{k})=\underset{i\in A_{k}}{\prod }f_{inc}(O_{i}-T_{i})$ was the probability that individual *i* ∈ *A_k_* (i.e. the primary cases) developed symptoms at time *O_i_* given that he/she was infected at time *T_i_*.
2. Let Λ*_j_*(*t*) be the force of infection that the primary cases exerted on individual *j* at time *t*, and φ be the probability of case ascertainment among those infected. If there was only one non-primary case in cluster *k*, then $L_{k,2}(\theta |T_{i},i\in A_{k})=\varphi \int_{T_{\text{min},j}}^{T_{\text{max},j}}\Lambda_{j}(T_{j})\exp\left(-\int_{0}^{T_{j}}\Lambda_{j}(t)\text{d}t\int_{0}\right)\;f_{\text{inc}}(O_{j}-T_{j})\text{d}T_{j}$ was the probability that the non-primary case *j* ∈ *B_k_* (i) was infected at time *T_j_* ∈ [*T*_min,*j*_, *T*_max,*j*_], (ii) developed symptoms at time *O_j_* and (iii) was ascertained. If there were more than one non-primary case in cluster *k* (i.e. some of them might be tertiary cases), then
  $$L_{k,2}(\theta |T_{i},i\in A_{k})=1-\underset{j\in B_{k}}{\prod }\left[1-\varphi \left(1-\text{exp}\left(-\int_{T_{min,j}}^{T_{max,j}}\Lambda_{j}(t)\;\text{d}t\right)\right)\right]$$
  was the probability that at least one (but not necessarily all) of the non-primary cases *j* ∈ *B_k_* was infected by the primary cases. The force of infection $\Lambda_{j}(t)=\sum_{i\in A_{k}}\lambda_{ij}(t)$ where *λ_ij_*(*t*) was the force of infection exerted by primary case *i* ∈ *A_k_* on individual *j* at time *t.* We modelled *λ_ij_*(*t*) as follows:
  $$\lambda_{ij}(t)=\omega (k)L(t)\beta (n_{k})\delta_{inf}(g_{i})\delta_{sus}(g_{j})\alpha (a_{j})f_{\text{gen}}(t-T_{i}|\theta ,O_{i}-T_{i})Q_{ij}(t),$$

where:

— *ω*(*k*) was the relative transmission rate in Anqing compared to Shenzhen, i.e. *ω*(*k*) = *ω_AQ_* if cluster *k* was from Anqing and 1 if it was from Shenzhen.
— *L*(*t*) was the relative transmission rate after Wuhan was locked down, i.e. *L*(*t*) = *σ* if time *t* was after 23 January 2020 and 1 otherwise.
— *β*(*n*) was the baseline transmission rate for clusters of size *n*.
— $\delta_{\text{inf}}(\cdot )$ was sex-specific infectiousness (assumed to be 1 for females).
— $\delta_{\text{sus}}(\cdot )$ was sex-specific susceptibility (assumed to be 1 for females).
— $\alpha_{\text{inf}}(\cdot )$ was age-specific infectiousness which was assumed to be (i) equal to 1 for those aged 40 years or above (this age group accounted for around half of all primary cases in our dataset; figure 1*b*); and (ii) a piece-wise cubic Hermite interpolating polynomial function (https://www.mathworks.com/help/matlab/ref/pchip.html) of age for those aged under 40 years where the value of *α*_inf_(0) was included in *θ* for statistical inference. In the sensitivity analysis, we considered age 30 and 50 as alternative age cutoffs (electronic supplementary material, figure S14).
— $\alpha_{\text{sus}}(\cdot )$ was age-specific susceptibility which was assumed to be (i) equal to 1 for those aged 60 years or above and (ii) a piece-wise cubic Hermite interpolating polynomial function (https://www.mathworks.com/help/matlab/ref/pchip.html) of age for those aged under 60 years where the value of $\alpha_{\text{sus}}(\cdot )$ at age 0, 15 and 30 years were included in *θ* for statistical inference.
— *Q_ij_*(*t*) = 1 if individual *j* was exposed to individual *i* at time *t* and 0 otherwise (see electronic supplementary material, figures S1–S13).

3. $L_{k,3}(\theta |T_{i},i\in A_{k})=\prod_{j\in C_{k}}\left[1-\varphi \left(1-\text{exp}\left(-\int_{0}^{\infty }\Lambda_{j}(t)\;\text{d}t\right)\right)\right]$ was the probability that all individuals in *C_k_* had not been ascertained to be infected by the end of the cluster outbreak.

The overall likelihood for cluster *k* was

$$L_{k}(\theta )=\int_{\Omega_{k}}L_{k}(\theta |T_{i},i\in A_{k})\underset{i\in A_{k}}{\prod }h_{\text{inf},i}(T_{i})\;\text{d}T_{i},$$

where Ω*_k_* was the Cartesian product of [*T*_min,*i*_, *T*_max,*i*_] for all *i* ∈ *A_k_* and *h*_inf,*i*_ was the pdf of the time of infection for individual *i* ∈ *A_k_*. Let *T*_lockdown_ denote the time when Wuhan was locked down (i.e. 23 January 2020). Given that (1) primary cases had history of travelling to or were residents of Hubei, or had contact history with suspected cases or returnees from Hubei; and (2) community transmission of COVID-19 in Shenzhen and Anqing was relatively low during the study time horizon, we assumed that the probability of *T_i_* = *t* was proportional to the epidemic size in Wuhan at time *t* if time *t* < *T*_lockdown_ and zero otherwise. That is, $h_{\text{inf},i}(T_{i})={\text{e}}^{rT_{i}}/\int_{T_{\text{min},i}}^{T_{\text{max},i}^{*}}{\text{e}}^{r\tau \;}\text{d}\tau =re^{rT_{i}}/({\text{e}}^{rT_{\text{max},i}^{*}\;}-{\text{e}}^{rT_{\text{min},i}})$ for *T_min_*_,*i*_ ≤ *t* ≤ $T_{\text{max},i}^{*}$ where *r* was the epidemic growth rate in Wuhan (assumed to be 0.133 day^−1^ which corresponded to a doubling time of 5.2 days [19]) and $T_{\text{max},i}^{*}=\text{min}(T_{\text{max},i},T_{\text{lockdown}})$.

To integrate published estimates of age-specific and sex-specific susceptibility into our inference, we used the estimates from Jing *et al*. [15] and Zhang *et al*. [16] as priors for age- and sex-specific susceptibility (see electronic supplementary material, table S3 for details). We used flat non-informative priors (uniform distributions on [0, ∞)) for the remaining parameters because there were little published or publicly available evidence on their values. We estimated the model parameters *θ* using Markov Chain Monte Carlo methods with Gibbs sampling. Point estimates and statistical uncertainty were presented using the maximum posteriori probability (MAP) estimate and 95% credible intervals (CrIs), respectively.

### (c) Age-structured transmission model

We partitioned the population of each jurisdiction into the following *m* = 15 age groups: 0–4, 5–12, 13–18, 19–23, 24–29, 30–34, 35–39, 40–44, 45–49, 50–54, 55–59, 60–64, 65–69, 70–74 and 75+. We assumed that age had no effect on infectiousness (which was consistent with the absence of strong evidence for age effect on COVID-19 infectiousness in figure 1*d*). Let *α_i_* be the relative susceptibility of age group *i*. For COVID-19, we parametrized *α_i_*'s according to figure 1*c*. For H1N1pdm09, we parametrized *α_i_*'s according to a previous household study which indicated those aged 0–18 and ≥50 were 2 and 0.2 times as susceptible compared with those aged 19–50 [24].

We used the following SIR model to simulate COVID-19 and influenza epidemics where *S_i_*(*t*) and *R_i_*(*t*) were the number of susceptible and recovered individuals in age group *i* at time *t*, and *I*(*t*, *τ*) was the number of infected individuals in age group *i* at time *t* who were infected at time *t* − *τ*:

$$\begin{array}{rl} \frac{\text{d}S_{i}(t)}{\text{d}t} & =-\alpha_{i}S_{i}(t)\pi_{i}(t),\\ \frac{\partial I_{i}(t,\tau )}{\partial t}+\frac{\partial I_{i}(t,\tau )}{\partial \tau } & =-f_{\text{gen}}(\tau )I_{i}(t,\tau ),\\ I_{i}(t,0) & =\alpha_{i}S_{i}(t)\pi_{i}(t),\\ \frac{\text{d}R_{i}(t)}{\text{d}t} & =\int_{0}^{t}f_{\text{gen}}(\tau )I_{i}(t,\tau )\;\text{d}\tau \\ \;\text{and}\;\pi_{i}(t) & =\sum_{j=1}^{m}\frac{\beta_{ij}}{N_{j}}\int_{0}^{t}I_{j}(t,\tau )\;\text{d}\tau , \end{array}$$

where *β_ij_* was the contact rate between age group *i* and *j* from Prem *et al.* [20] and *N*_1_, … , *N_m_* was the age distribution obtained from World Population Prospects 2019. (https://population.un.org/wpp/DataQuery/).

The next-generation matrix (NGM) for this SIR model was

$$T_{G}\left[\begin{array}{ccc} \alpha_{1}\beta_{11} & \cdots & \alpha_{1}\beta_{1m}\\ \vdots & \ddots & \vdots \\ \alpha_{m}\beta_{m1} & \cdots & \alpha_{m}\beta_{mm} \end{array}\right],$$

where *T_G_* was the mean generation time (figure 1*e*). The basic reproductive number *R*_0_ was the largest eigenvalue of this matrix.

The synthetic contact matrices from Prem *et al.* [20] could be decomposed by the settings in which contacts were made: household, primary school or below, secondary school or above, workplace and community. Let *y_ijs_* be the average number of contacts that an individual from age group *i* made with age group *j* in setting *s* in the synthetic contact matrices. The corresponding average number of contacts between age groups *i* and *j* in setting *s* was *N_i_y_ijs_*, and the proportion of contacts made in setting *u* compared to all contacts made was $\sum_{i\leq j}N_{i}y_{iju}/\sum_{s}\sum_{i\leq j}N_{i}y_{ijs}$.

In our model, if school closure was implemented, all contacts made at school were removed from the contact matrix (unless specified otherwise) and the resulting NGM was used to calculate the reproductive number. Similarly, when the frequency or transmissibility of contacts made in a given setting (e.g. community) was reduced by *x*%, the contact matrix of that setting was discounted by *x*% accordingly.

## 3. Results

### (a) Household transmission dynamics

We estimated that children were substantially less susceptible to COVID-19 infection than adults and the elderly (figure 1*c*). Specifically, individuals aged 0–12 (primary school age and below), 13–23 (secondary school and higher education age) and 24–59 (adults) were 0.23 (0.15–0.33), 0.28 (0.13–0.45) and 0.68 (0.58–0.85) times as susceptible as those aged 60 or above, respectively. We found that children were less susceptible to COVID-19 infection primarily because of the lower attack rate observed among children in our dataset (figure 1*a*; electronic supplementary material, table S1). Given that only 15 primary cases were younger than 20 and they were co-primary cases with their parents or caregivers in our data (figure 1*b*), the posterior distribution of the relative infectiousness of children was not sufficiently precise to refute the null hypothesis that children and adults were equally infectious (figure 1*d*). The mean generation time and serial interval was 4.8 (4.0–5.9) days and 7% of serial intervals were negative (figure 1*e*). Infectiousness peaked when infected individuals were 44% (10–66%) through their incubation period. Consequently, 67% (56–78%) of infections occurred before the infectors developed symptoms. Compared with females, males were 0.86 (0.75–0.97) times as susceptible to infection (figure 1*f*). We estimated that males were 1.31 (0.41–1.92) times more infectious than females, i.e. weak evidence that the difference in infectiousness between males and females (if any) was significant (figure 1*f*). Within-household transmissibility was similar between Shenzhen and Anqing and dropped by 62% (11–86%) after lockdown measures were implemented in Wuhan on 23 January followed by all major Chinese cities shortly after (figure 1*f*). These inference results were insensitive to our assumptions regarding the incubation period (mean 5.5 days with a coefficient of variation 0.4 in the base case) except that the proportion of presymptomatic transmission would increase (decrease) by around 10% if the mean incubation period was decreased (increased) by 1 day (electronic supplementary material, figure S14). The results were also insensitive to assumptions regarding the probability of case ascertainment among household contacts (0.7–1) and age cutoff for age-specific infectiousness (electronic supplementary material, figure S14).

### (b) School closure model

**Figure 2.**
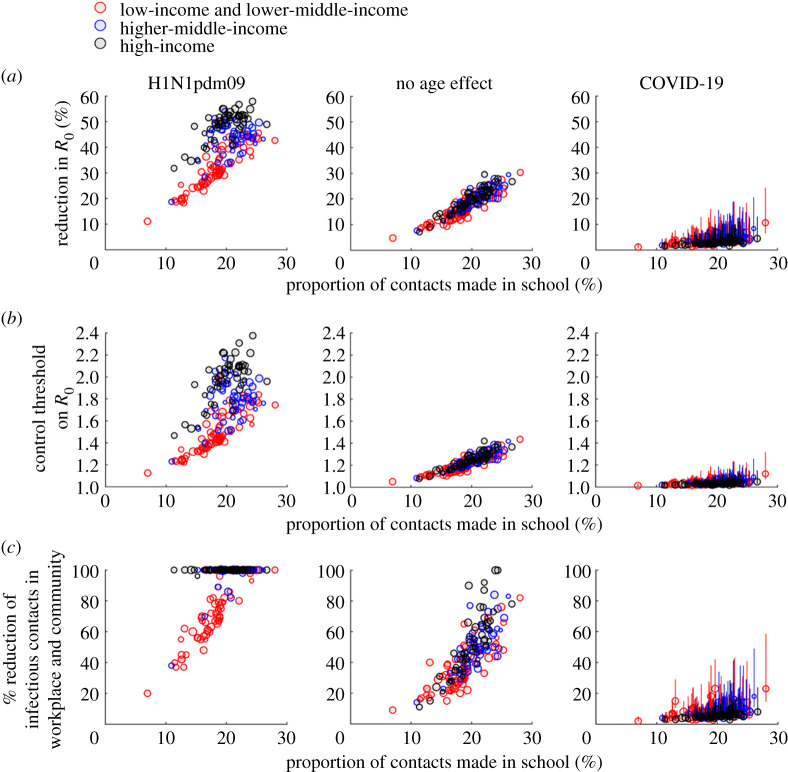
The effectiveness of school closure in reducing transmissibility as a function of proportion of contacts made in school across 177 jurisdictions. See electronic supplementary material, table S4 for the numerical values of all data points shown here. The sizes of data points are proportional to the log of the corresponding population size. Circles and bars (for COVID-19) indicate point predictions and 95% prediction intervals, respectively. (*a*) Reduction in *R*_0_ conferred by school closure against H1N1pdm09 (left), a pathogen to which individual of all ages were equally susceptible (middle) and COVID-19 (right). (*b*) Outbreak threshold (in terms of *R*_0_) under school closure. (*c*) Per cent reduction in workplace and community contacts that would be required in order to achieve the reduction in *R*_0_ conferred by school closure. A required percentage of 100% means that this was impossible. (Online version in colour.)

For COVID-19, we parametrized the transmission model with the posterior estimates of age-specific susceptibility from our within-household transmission inference and assumed that age had no effect on infectiousness (figure 2*c*,*d*). For pandemic influenza, we used the H1N1pdm09 parameters from a previous household transmission study which indicated those aged 0–18 and greater than 50 were 2 and 0.2 times as susceptible compared with those aged 19–50 [24]. We also considered seasonal influenza for which age had no clear effect on susceptibility or infectiousness.

To avoid underestimating the impact of school closure, we assumed (unless specified otherwise) that all contacts made at school would be eliminated during school closure and holidays while contacts in all other settings were unchanged. We estimated the reduction in basic reproductive number (*R*_0_) conferred by school closure in each jurisdiction; the reduction in effective reproductive number (*R*_t_) would be similar if the age distribution of the susceptibles was similar to the overall age distribution (e.g. when most of the population were still susceptible). We estimated the peak prevalence and IAR in each jurisdiction with and without school closure for different values of *R*_0_ (which reflected the baseline transmission dynamics when school closure started, i.e. the combined effect of the intrinsic transmissibility of the virus and the control measures in the population).

### (c) Effectiveness of school closure

The relative reduction in *R*_0_ conferred by school closure was primarily driven by the proportion of contacts made in school (figure 2*a*). Our results were consistent with previous studies that had empirically inferred the reduction of the reproductive number for influenza during school closure and holidays in mainland China, France, Hong Kong SAR, India, South Korea and the UK if 60% of school contacts were eliminated during these times while contacts in all other settings were unchanged (table 1).

Because schoolchildren were twice as susceptible to H1N1pdm09 compared with adults, school closure could generally reduce the *R*_0_ of H1N1pdm09 by 20–58% (except for South Sudan where the out-of-school rates exceed 60%; figure 2*a*). By contrast, school closure was much less effective at reducing the *R*_0_ of COVID-19, to which children were less susceptible compared with adults (figure 1*c*), even if all school contacts would be eliminated during school closure. Specifically, school closure could only reduce the *R*_0_ of COVID-19 by *x_R_* = 2–5%, 2–8% and 1–11% in high-income, upper-middle-income and other populations, respectively (figure 2*a* for the sensitivity of these estimates to the parametric uncertainty in age-specific susceptibility). This implies that school closure alone would be unable to control COVID-19 in these populations unless the reproductive number (that corresponded to the baseline contact matrix) was already below 1/(1 − *x_R_*) ≈ 1 + *x_R_*, i.e. close to the critical value of 1 (figure 2*b*). If only kindergartens and primary schools were closed, *R*_0_ of COVID-19 in high-income and other populations would be reduced by 1–2% and 1–5%, respectively. Closing secondary schools and higher education institutions would have similar effects except in low-income populations with high out-of-school rates among adolescents and youth of upper secondary school age or above. Comparable reductions in *R*_0_ conferred by school closure could be achieved by reducing infectious contacts in workplace and community by 3–9% and 2–23% for high-income and other populations, respectively (figure 2*c* for uncertainty levels). This might be achieved by reducing the frequency or transmissibility (e.g. face mask wearing and heightened personal hygiene) of these contacts. By contrast, this substitution strategy would be too disruptive to be practical or simply impossible for H1N1pdm09 (i.e. the effect of school closure could not be substituted even if all workplace and community contacts were to be eliminated).

**Figure 3.**
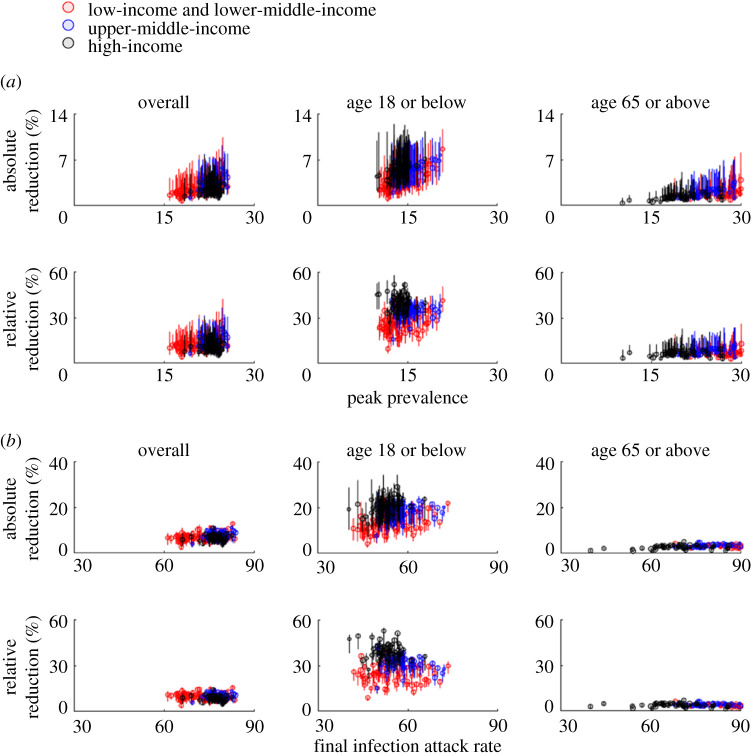
The public health impact of school closure across 177 jurisdictions when *R*_0_ = 2.5. See electronic supplementary material, table S4 for the numerical values of all data points shown here. Circles and bars indicate point predictions and 95% prediction intervals, respectively. (*a*) Reduction in peak prevalence for all ages (left), those aged 18 or below (middle) and those aged 65 or above (right). (*b*) Reduction in final IAR for all ages (left), those aged 18 or below (middle) and those aged 65 or above (right). (Online version in colour.)

**Figure 4.**
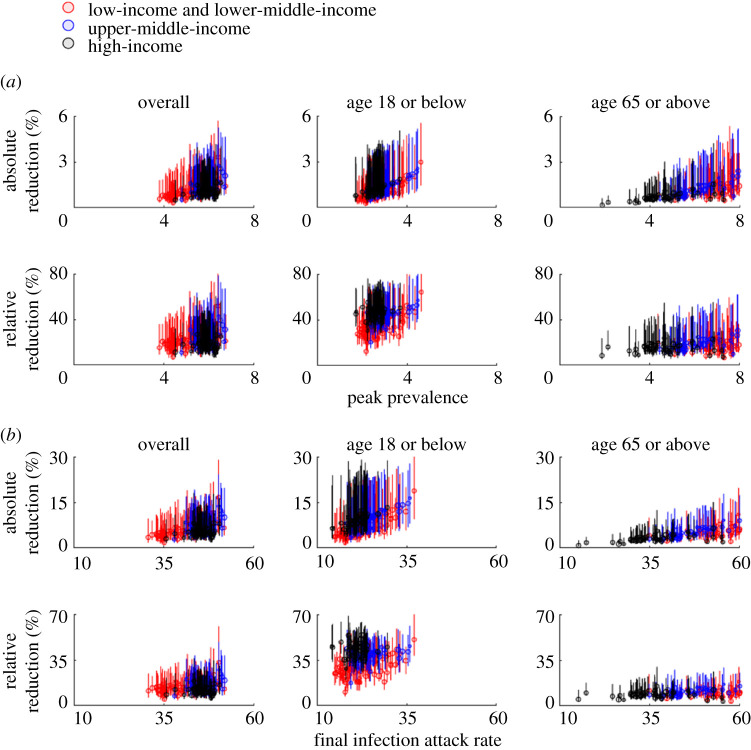
The same layout as in figure 3 when *R*_0_ = 1.5. (Online version in colour.)

For COVID-19, the reduction in morbidity and mortality conferred by school closure would depend on the baseline *R*_0_ as well as the timing and duration of school closure (figure 3). If the baseline *R*_0_ was around 2.5 (e.g. the epidemic was largely unmitigated), implementing school closure throughout the epidemic would reduce peak prevalence in high-income and other populations by 5–17% and 4–28%, respectively (figure 3*a* for uncertainty levels). The reductions would be higher for school children (because they are the target group of school closure) and slightly lower for older adults (because physical interactions among children and the elderly are relatively weak in general). By contrast, if the baseline *R*_0_ was around 1.5 (e.g. school closure was implemented as a complementary measure in addition to other NPIs such as the use of facemasks), implementing school closure throughout the epidemic would reduce peak prevalence by 9–27% and 7–52% in high-income and other populations, respectively (figure 4*a* for uncertainty levels). The reduction in final IAR would be lower than that for peak prevalence. For example, the overall IAR would be reduced by 6–19% and 5–33% in high-income and other populations if *R*_0_ = 1.5 (figure 4*b*); the corresponding reductions were 5–14% and 4–16% if *R*_0_ = 2.5 (figure 3*b*). Reducing infectious contacts in workplace and community settings by 3–28% would achieve the reduction in IAR under 18 conferred by school closure if *R*_0_ = 1.5; the corresponding reduction requirement would increase to 10–44% if *R*_0_ = 2.5.

Note that these comparative outcomes across jurisdictions were conditional on the baseline *R*_0_ which would not necessarily be the same in all populations due to heterogeneity in age structures, contact patterns, climatic factors and intervention policies. Importantly, our results do not suggest that prevalence and IAR would generally be higher or lower in any particular population. By the same token, our results do not suggest that school closure would generally confer greater reductions in peak prevalence and IAR in any particular population.

## 4. Discussion

The fundamental motivation for school closure presumes schoolchildren are a core transmission group. In concordance with recent studies [14–16], we have shown that this is not the case for COVID-19 because compared to older individuals, school children are substantially less susceptible to COVID-19. Consequently, even prolonged school closure that could hypothetically eliminate all school contacts for weeks to months would have limited effectiveness in reducing transmissibility.

Our results, together with recent findings that clinical severity of COVID-19 in children is lower than that for adults [19,25] and that prolonged school closure could significantly impair the emotional, mental and physical health of schoolchildren (see below) suggest that school closure may not be ideal as a sustained primary NPI for controlling COVID-19. In fact, the specific objective underlying school closure should be more clearly articulated so that alternative options could be comparatively assessed. For example, the overall disease transmission in the population could be mitigated by reducing infectious contacts (e.g. by enhancing personal infection prevention) which is likely to be more feasible and less disruptive to children's overall wellbeing than school closure (https://www.cdc.gov/coronavirus/2019-ncov/community/schools-childcare/operation-strategy.html). On the other hand, short-term school suspension could and should be considered when other NPIs (e.g. avoidance of mass gatherings, physical distancing in workplace and community, enhanced personal hygiene, etc.) are already in place, where the marginal effect of school closure is critical to achieving the target control levels (e.g. when hospitals have exceeded their surge capacity and clinical outcomes including the case-fatality risk are on the verge of unnecessarily deteriorating simply because the health system is about to implode).

Recent studies have highlighted numerous downsides of prolonged school closure on the mental and physical wellbeing of children, including increased risks of loneliness, academic achievement gaps, reduction in physical activity, malnutrition (for children who rely on school meals), increased risk of domestic abuse and violence, etc. [26–30]. Even within high-income populations, these adverse effects would likely be disproportionately amplified among socio-economically disadvantaged groups (e.g. lack of or limited access to affordable childcare, technical and financial support for digital learning, online social interactions and physical exercises). In addition, closing schools could inadvertently cause childcare shortages and hence worker absenteeism among parents. The resulting worker absenteeism in the healthcare system (which has been estimated to be around 20% for the US healthcare workforce) could indirectly exacerbate the case-fatality risk of COVID-19 patients and therefore the overall mortality rate of the pandemic due to weakened healthcare [31]. As such, compensatory measures should be proactively devised to ameliorate these adverse events if school closure is employed to control COVID-19. For example, the childcare arrangement should be explicitly considered in order to minimize unintended worker absenteeism and the downstream adverse effect on healthcare quality.

We estimated that within-household transmissibility in Shenzhen and Anqing was reduced by 62% (11–86%) in January–March after nationwide lockdown measures were implemented in China. Given that human mobility in all Chinese major cities was reduced by greater than 90% during this period (all individuals were mandated to stay home and only one household member was allowed to leave home every 2 to 3 days to procure basic (e.g. food) and essential (e.g. medications) supplies, such reduction in within-household transmissibility was likely due to heightened precautions of personal infection prevention. Sustained adoption of such precautions in the 177 jurisdictions could in principle reduce the reproductive number of COVID-19 by 6–41%, i.e. more effective than school closure.

We estimated that compared with females, males were less susceptible to COVID-19 infection but might be more infectious (albeit the uncertainty in our estimate of the latter is high; figure 1*f*). Our estimates are consistent with that from the largest household transmission study in China [32]. However, seroprevalence studies from other countries suggested that males might be more susceptible to infection than females [33,34]. As such, further investigations are needed to elucidate the effect of sex on susceptibility to and infectiousness of COVID-19 infection.

Our study has several important limitations. First, we assumed that interactions, case ascertainment and infection prevention measures were homogeneous among members within households, hence attributing the effect of age on the risk of COVID-19 infection to intrinsic biological susceptibility. In reality, adult–adult and adult–child interactions might be associated with different transmission rates, and case ascertainment and infection prevention measures might be age-dependent (though less likely given that the whole country implemented strict lockdowns at maximal pandemic response level). Furthermore, during the 2009 influenza pandemic, household transmission models similar to ours generated age-specific susceptibility estimates for H1N1pdm09 [23,24] that were consistent with that from community transmission and sero-surveillance models [7]. Taken together, these studies provide external support for our surmise that children were biologically less susceptible to COVID-19 infection and our estimates of age-specific susceptibility are applicable in general transmission settings.

Second, we assumed that our estimates based on data from Chinese cities were applicable to the rest of the world. While our estimates are consistent with similar results from other localities [14,35–37], more region-specific data would be needed to validate this assumption.

Third, we assumed that school closure would eliminate all school contacts without affecting contacts in other settings. In reality, school closure could increase some non-school contacts (e.g. more contacts between children and their caregivers, more contacts among children outside schools), in which case school closure against COVID-19 would be less effective than estimated here. This is particularly true in resource-constrained settings where childcare assistance and personal protective measures (e.g. facemasks and hand hygiene supplies) are limited.

Fourth, we assumed that age had no effect on infectiousness [38]. If children are less infectious than adults, as postulated by recent studies [39], then school closure would be less effective than estimated here.

Fifth, our framework is built on the contact matrices synthesized by Prem *et al*. [20] which have been increasingly used to study infectious diseases including COVID-19 [14,40,41]. These have been compared against empirical surveys, in terms of both raw numbers of contacts and impact of interventions in COVID-19 models. Comparisons indicate good concordance between empirical and synthetic matrices [20]. However, differences are observed in older adults in some countries, which could be partly due to small sample sizes in older adults in empirical studies, particularly in countries with younger populations. This highlights the critical importance of conducting periodic sufficiently powered social contact surveys in different parts of the world for informing epidemic surveillance and control of a wide range of infectious diseases including COVID-19, influenza, TB, RSV, measles, etc.

Sixth, almost all contact studies (whether empirical or synthetic) define contacts as involving either physical touch or two-way conversations (see e.g. Mossong *et al*. [42]). Although students may be in a class of 20 other students, they only have ‘contact’ with a small subset of them. Indeed, studies have found that when contacts are defined as such, there is no statistically significant association between class size and total number of contacts (see e.g. Béraud *et al*. [43]). This matches guidelines and current (limited) understanding of SARS-CoV-2 transmission; for instance the United States Centers for Disease Control and Prevention define a ‘close contact’ (mandating quarantine) as someone who was ‘within 6 feet of someone who has COVID-19 for a total of 15 min or more’ (https://www.cdc.gov/coronavirus/2019-ncov/if-you-are-sick/quarantine.html). However, it cannot be ruled out that transmission can occur between classmates who are not close contacts. This highlights the importance of evidence-based school opening guidelines, such as minimizing movement within the classroom, keeping desks well space, encouraging face covering and frequent handwashing and ensuring good ventilation in classrooms whenever practicable.

## Data accessibility

All data are available in the main text or electronic supplementary material. Codes are available in the GitHub repository: https://github.com/kathyleung/2020_08_school_closure. Readers without access to Matlab can run the scripts on our servers by contacting the corresponding author for account registration.

The data are provided in the electronic supplementary material [44].
